# Synergistic inhibition of tumor cell proliferation by metformin and mito-metformin in the presence of iron chelators

**DOI:** 10.18632/oncotarget.26943

**Published:** 2019-05-28

**Authors:** Gang Cheng, Jacek Zielonka, Micael Hardy, Olivier Ouari, Christopher R. Chitambar, Michael B. Dwinell, Balaraman Kalyanaraman

**Affiliations:** ^1^Department of Biophysics, Medical College of Wisconsin, Milwaukee, WI 53226, USA; ^2^Free Radical Research Center, Medical College of Wisconsin, Milwaukee, WI 53226, USA; ^3^Cancer Center, Medical College of Wisconsin, Milwaukee, WI 53226, USA; ^4^Department of Medicine, Medical College of Wisconsin, Milwaukee, WI 53226, USA; ^5^Department of Microbiology and Immunology, Medical College of Wisconsin, Milwaukee, WI 53226, USA; ^6^Department of Surgery, Medical College of Wisconsin, Milwaukee, WI 53226, USA; ^7^Aix Marseille University, CNRS, ICR, UMR 7273, Marseille 13013, France

**Keywords:** iron chelators, mitochondria-targeting agents, cancer cell proliferation, biguanide; metformin analogs

## Abstract

We demonstrate that combined treatment with metformin (Met) or its mitochondria-targeted analog, mito-metformin (Mito-Met), and various iron chelators synergistically inhibits proliferation of pancreatic and triple-negative breast cancer cells. Previously, we reported that Met (at millimolar levels) and Mito-Met (at micromolar levels) inhibited pancreatic cancer cell proliferation. Inhibition of mitochondrial complex I and mitochondrial oxidative phosphorylation (OXPHOS) through stimulation of mitochondrial redox signaling was proposed as a key mechanism for decreased cancer cell proliferation. Because of its poor bioavailability, the use of Met as a “stand-alone” antitumor drug has been questioned. Iron chelators such as deferasirox (DFX) and dexrazoxane (DXR) exhibit antiproliferative effects in cancer cells. The potency of Met and Mito-Met was synergistically enhanced in the presence of iron chelators, DFX, N,N'-bis(2-hydroxyphenyl)ethylendiamine-N,N'-diacetic acid (HBED), and deferoxamine (DFO). Met, DXR (also ICRF-187), and DFO are FDA-approved drugs for treating diabetes, decreasing the incidence and severity of cardiotoxicity following chemotherapy, and mitigating iron toxicity, respectively. Thus, the synergistic antiproliferative effects of Met and Met analogs and iron chelators may have significant clinical relevance in cancer treatment. These findings may have implications in other OXPHOS-mediated cancer therapies.

## INTRODUCTION

**Figure 1:**
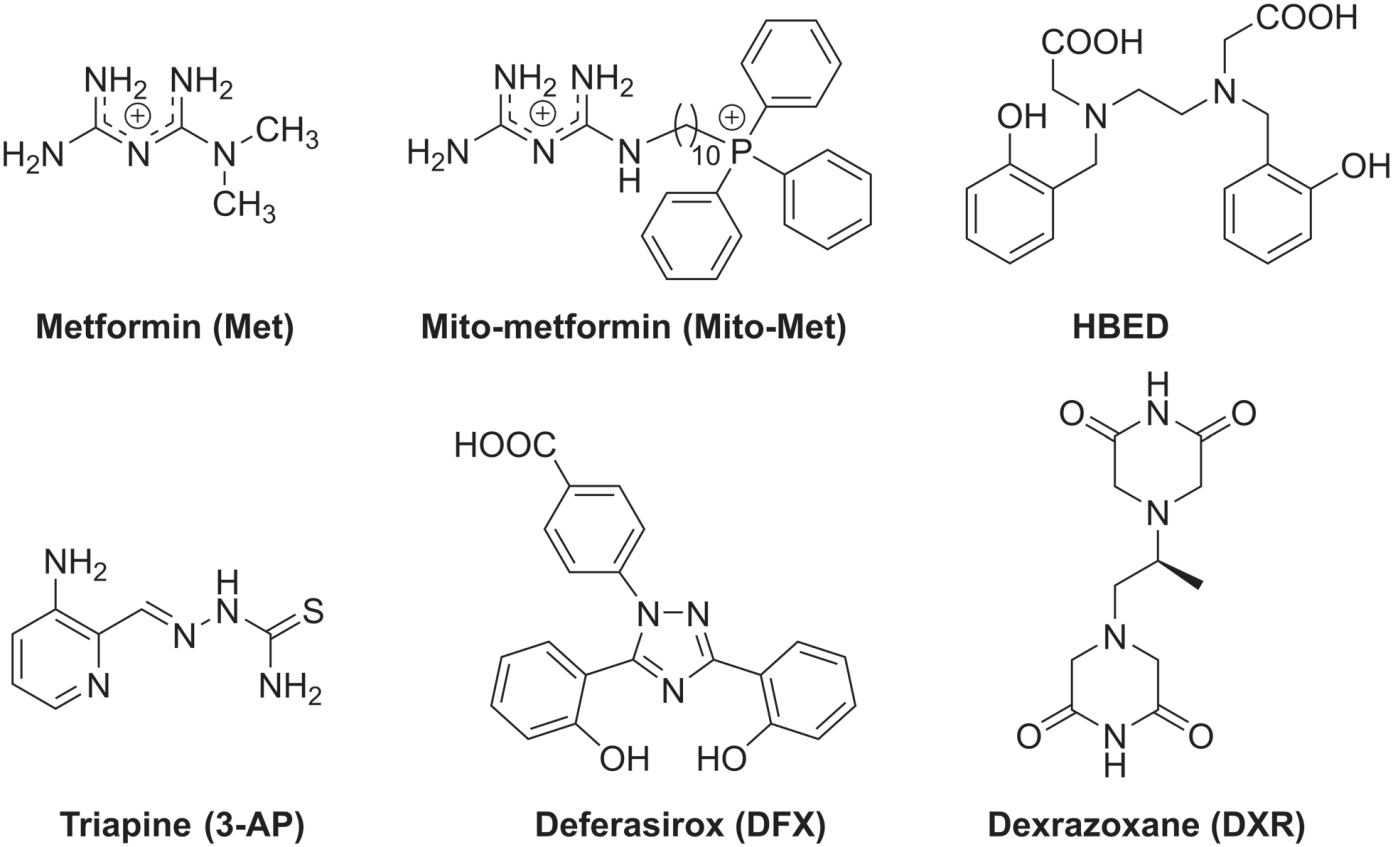
Chemical structures of iron chelators, Met, and Mito-Met used in this study.

Metformin (Met), a synthetic biguanide analog of guanidines found in *Galega officinalis* and an FDA-approved drug, is one of the most widely used antidiabetic drugs (Figure 1) [1]. Although the bioavailability of Met is poor, it has a very good safety profile, and diabetic patients typically tolerate daily doses of gram quantities of the drug [2]. Recent studies suggest that diabetic patients taking Met exhibit a decreased incidence of pancreatic cancer [3, 4]. Several clinical trials are currently underway exploring the possibility of repurposing Met as a potential antitumor drug in other cancers [5, 6]. A prevailing view is that Met targets mitochondria, albeit weakly; inhibits complex I in the mitochondrial electron transport chain; and activates the “AMPK/mTOR pathway” involved in regulating cellular metabolism, energy homeostasis, and cell growth [7, 8]. Although Met is relatively safe, the plasma concentration reaches only a few micromolar, even at high doses (500–1,000 mg/day), in humans. This raises a concern about the therapeutic feasibility for Met to act as an effective antitumor agent. There is a critical need to enhance the antitumor potency of Met through combinatorial drug therapy. To this end, analogs of Met (Mito-Met) conjugated to varying alkyl chain lengths containing a triphenylphosphonium cation (TPP^+^) were synthesized and characterized [7]. The Mito-Met analog (*e.g.*, Mito-Met shown in Figure 1) was nearly 1,000 times more effective than Met in inhibiting pancreatic cancer cell proliferation *in vitro* and tumor progression *in vivo* [7].

Both Met and Mito-Met exert a potent radiosensitizing effect in tumor cells [7, 9, 10]. Mito-Met was significantly more effective than metformin in enhancing cancer cell radiosensitivity [7]. Iron chelators induce an antiproliferative effect in tumor cells by causing cell cycle arrest [11]. Iron chelators with high antiproliferative activity also upregulate the expression of a tumor suppressor gene [12]. Thus, we postulated that combining iron chelators with mitochondria-targeted drugs (*e.g.*, Met or Mito-Met) will induce synergistic antiproliferative effects in cancer cells. Tumor cells have different levels of hypoxic areas, with the central core exhibiting maximal hypoxia [13, 14]. However, most *in vitro* experiments on cancer cells are performed under normoxic conditions, and the results obtained under such conditions may be different from results from the same experiments conducted at lower oxygen tensions. Several FDA-approved iron chelators including deferoxamine (DFO), a hexadentate chelator, and deferasirox (DFX), a tridentate chelator (Figure 1), target both proliferating and quiescent cells [15–17]. Thus, the potential for clinical translation of the combined use of Met and iron chelators in cancer treatment is high. In this study, we report that treatment of pancreatic and triple-negative breast cancer cells with Met and Mito-Met and selected structurally different iron chelators exerts synergistic antiproliferative effects. Because some of these compounds are FDA-approved and orally effective drugs, their clinical application in cancer treatment is possible.

## RESULTS

### Inhibition of pancreatic cancer cell proliferation by iron chelators and metformin analogs

**Figure 2:**
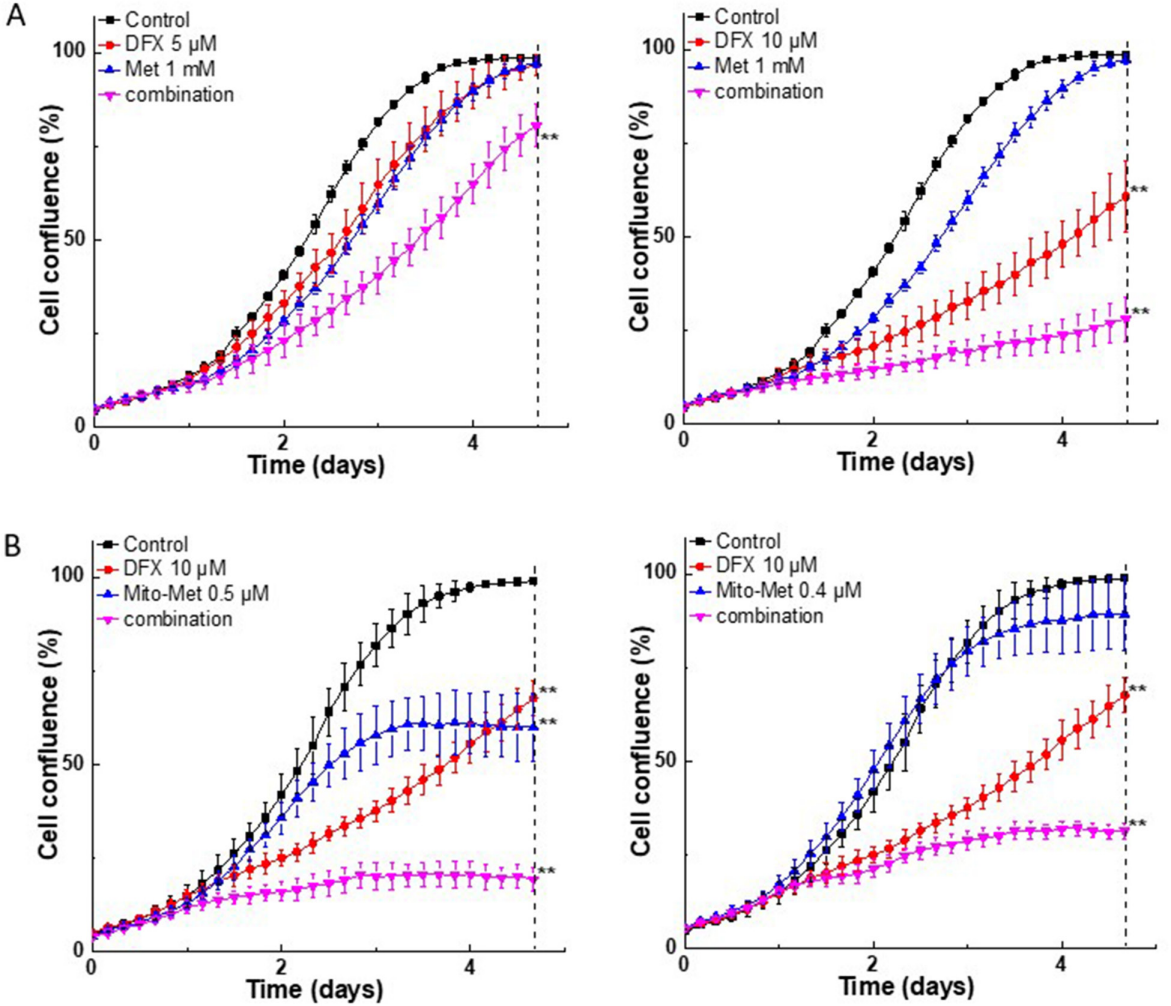
Effect of Met, Mito-Met, and iron chelator, DFX, on MiaPaCa-2 cell proliferation. Cells were treated with DFX (5 or 10 μM) and Met (**A***, left and right*) or Mito-Met (**B***, left and right*) independently and together, as indicated, and cell growth was monitored continuously. Data shown are the mean ± SD (*n* = 4). The dotted vertical lines indicate the time points at which the level of significance was calculated (^**^P < 0.01).

We determined the antiproliferative effects of the combination of Met or Mito-Met with structurally different chelators: DFX, an orally available iron chelator used for treatment of iron overload; dexrazoxane (DXR), which protects against doxorubicin-induced cardiotoxicity; and 3-AP (also called Triapine), an experimental anticancer drug and a potent inhibitor of ribonucleotide reductase. Figure 2 shows the antiproliferative effect of DFX and Met or Mito-Met in MiaPaCa-2 cells. The strongest antiproliferative effects were observed using the combination of Met or Mito-Met with the DFX chelator. Next, we investigated the combinatorial effects of Met or Mito-Met in the presence of DXR in PANC-1 cell proliferation. Again, the strongest antiproliferative effects were detected for Mito-Met and iron chelator (Supplementary Figure 1). Strong potentiation of antiproliferative effects of metformin by iron chelators was also observed in the case of AsPC-1, a second human pancreatic cancer cell line (Supplementary Figure 2), and FC-1242, a murine pancreatic cancer cell line isolated from spontaneous KRAS-p53 mutant pancreatic tumors [18] (Supplementary Figure 3).

### Synergistic antiproliferative effect of iron chelators and Met in breast cancer cells

**Figure 3:**
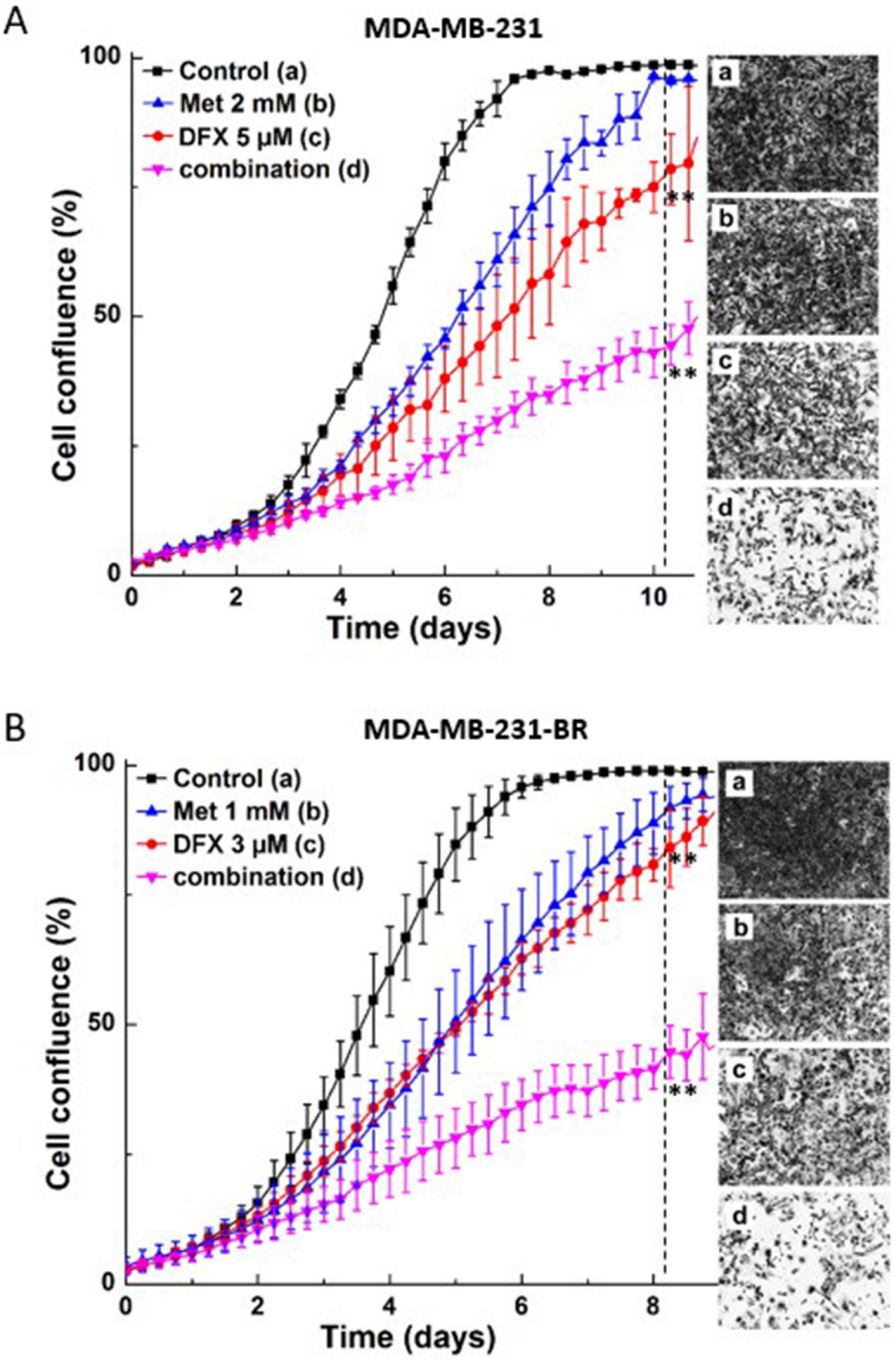
Effect of Met and iron chelator, DFX, on MDA-MB-231 and MDA-MB-231-BR cell proliferation. Cells were treated with DFX or Met independently and together, as indicated; MDA-MB-231 (**A**) and MDA-MB-231-BR (**B**) cell growth was monitored continuously. Data shown are the mean ± SD (*n* = 4). The dotted vertical lines indicate the time points at which cell pictures were taken and the statistical significance was calculated (^**^P < 0.01).

We determined the synergistic antiproliferative effects of the iron chelator, DFX, and Met on breast cancer cells. Triple-negative breast cancer cells, MDA-MB-231, and brain homing breast cancer cells, MDA-MB-231-BR, were treated with Met or DFX alone and together. Cell proliferation was monitored in real time and showed that the combination of DFX and Met inhibits more than additively (Figure 3). Other iron chelators (*e.g.*, N,N’-bis(2-hydroxyphenyl)ethylendiamine-N,N’-diacetic acid [HBED]) dose-dependently decreased the survival fraction of MDA-MB-231 breast cancer cells (not shown).

### Quantitative analyses of the synergy between iron chelator, HBED, and metformin analogs in the inhibition of MiaPaCa-2 cell proliferation

**Figure 4:**
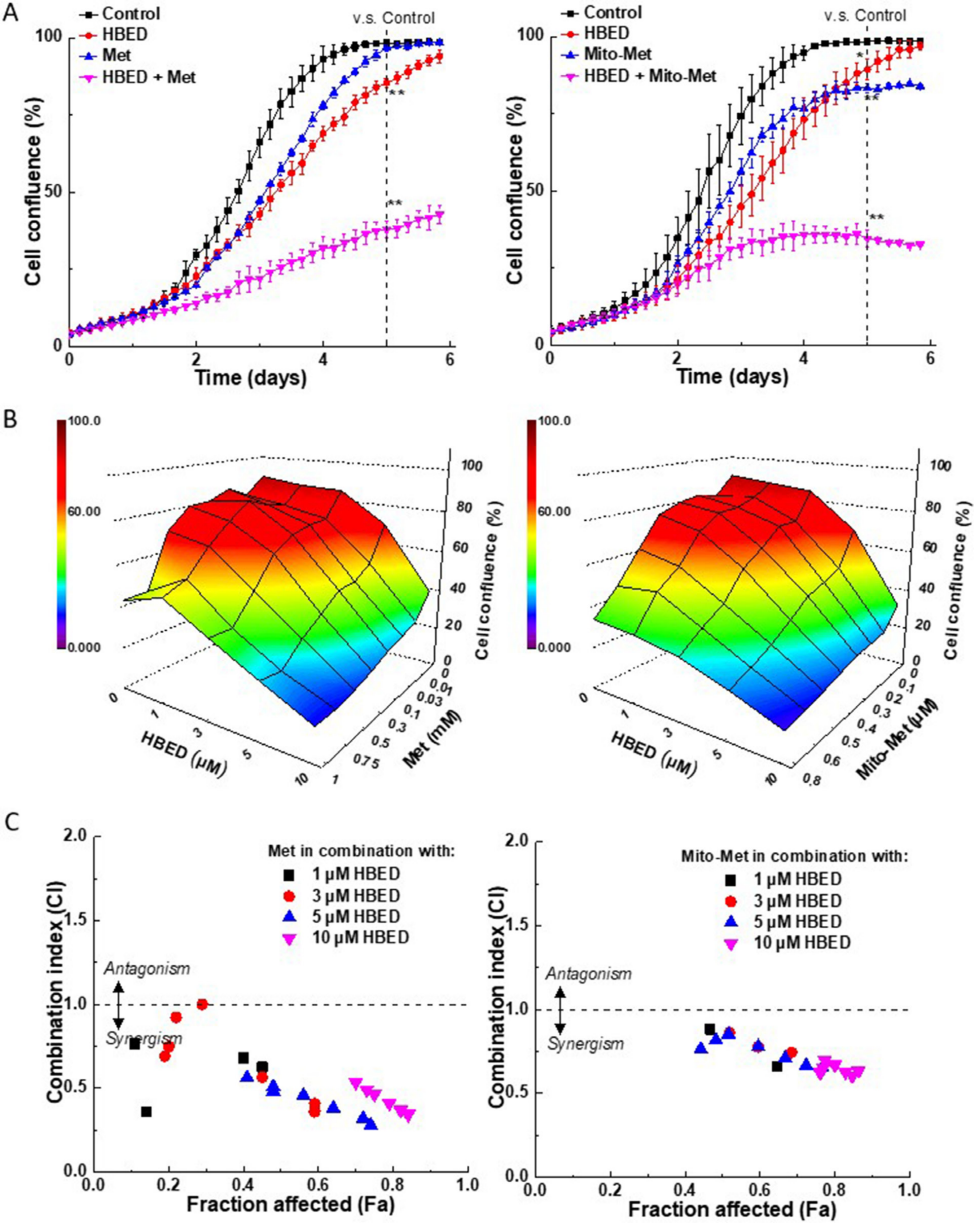
Synergy analyses of the effect of the combination of iron chelator, HBED, with Met, or Mito-Met on MiaPaCa-2 cell proliferation. (**A**) MiaPaCa-2 cells were treated with HBED (5 μM) or Met (0.5 mM, *left*), Mito-Met (0.4 μM, *right*) independently and together and cell growth monitored continuously. Data shown are the mean ± SD (*n* = 3). The dotted vertical lines indicate the time points at which the levels of significance were calculated (^**^P < 0.01). (**B**) Effects of Met (*left*) and Mito-Met (*right*) on cell proliferation. The cell confluence (as control groups reach 90% confluency) is plotted as a three-dimensional representation showing the concentration-dependent effects of HBED, Met, or Met analog alone and together on cell proliferation. Panel (**C**) shows the CI-Fa plots. The fraction affected parameter is used as a measure of the drug’s efficiency, with a value of one indicating complete inhibition of cell confluence and a value of zero indicating a lack of effect on cell confluence.

We monitored the effect of the compounds studied on the proliferation of MiaPaCa-2 pancreatic cancer cells in real time using the IncuCyte Live-Cell Imaging system. Figure 4A shows the antiproliferative effects of Met (*left*) and Mito-Met (*right*) and the iron chelator, HBED, either alone or in combination in pancreatic cancer cells, MiaPaCa-2. At the concentration of the compounds, which had only modest effects when used alone, the combination of Met or Mito-Met with HBED chelator led to stronger antiproliferative effects. Next, we investigated the concentration-dependent effect of the combination of the above compounds on cell proliferation. The cell confluence (as control groups reach 90% confluency) was plotted against the concentrations of the drugs. Figure 4B shows a three-dimensional heat map representation of the dependence of cell confluence on the concentrations of Met and HBED (*left*) and Mito-Met and HBED (*right*), alone and in combination. Figure 4C depicts the combination index-fraction affected (CI-Fa) plots. The CI parameter is a measure of the synergy, with values below one indicating synergy, a value of one indicating additive effects, and values above one indicating drug antagonism. The Fa parameter is used as a measure of the drug’s efficacy, with a value of one indicating total inhibition of cell proliferation and a value of zero indicating the lack of effect of the treatment on cell confluence. As shown in Figure 4C, both Met *(left*) and Mito-Met (*right*) exert synergistic inhibition of cell proliferation at several concentrations (1–10 μM) of HBED.

### Effect of Met and Mito-Met on colony formation by MiaPaCa-2 cells

**Figure 5:**
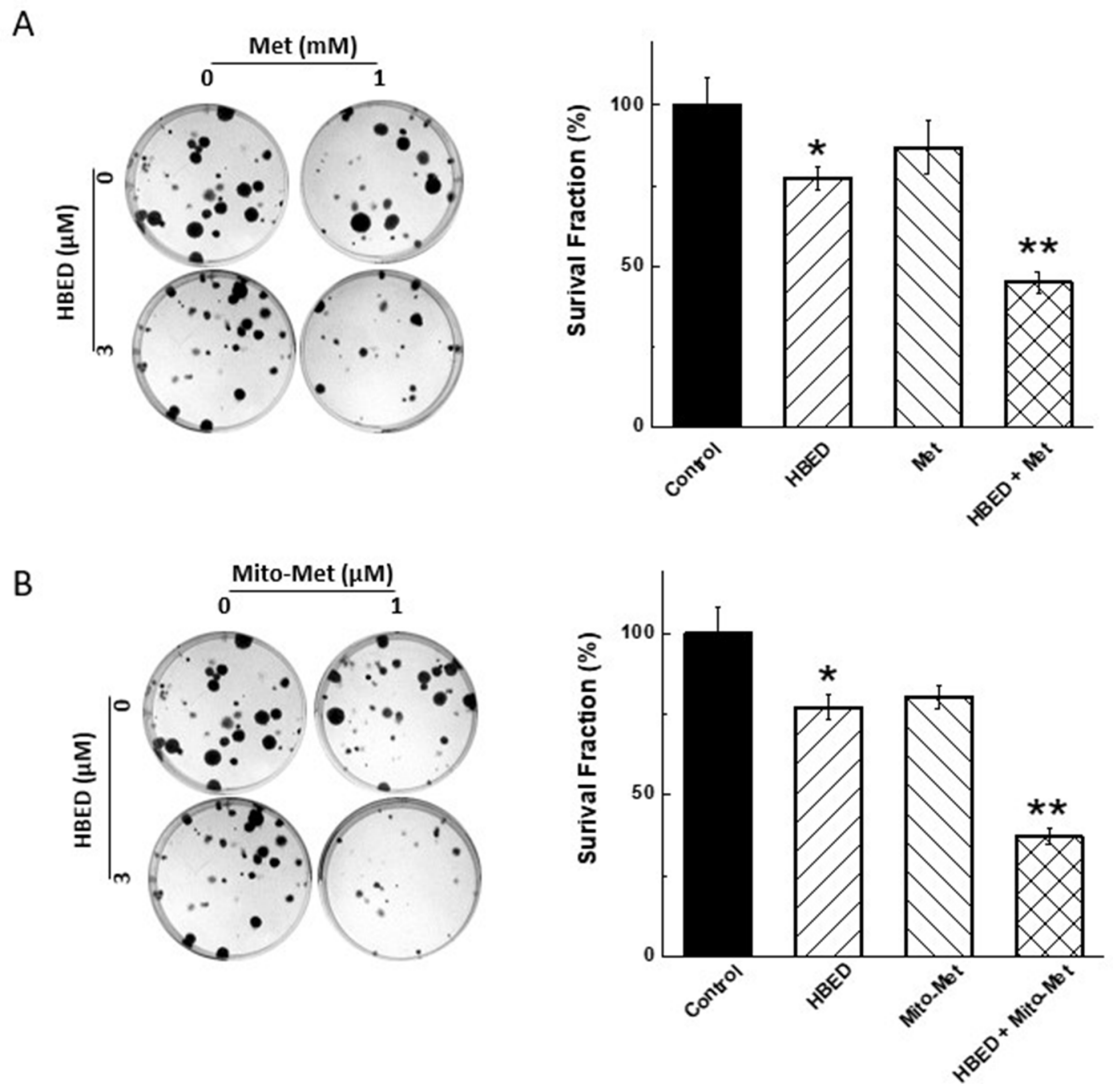
Effect of Met, Mito-Met, and HBED on colony formation by MiaPaCa-2 cells. (**A**) MiaPaCa-2 cells were treated with HBED (3 μM), Met (1 mM) alone or together, the colony formation was monitored (*left*), the number of colonies counted, and the survival fraction calculated (*right*). (**B**) Same as in (A), but Mito-Met (1 μM) was used instead of Met. Data shown represent the mean ± SEM. ^*^P < 0.05 (*n* = 6) comparing the HBED/Mito-Met or HBED/Met combination to HBED alone.

MiaPaCa-2 cells were treated with Met or Mito-Met in the presence of HBED, and the number of colonies formed was counted. As shown in Figure 5A (*left*), the number of colonies formed in the presence of Met and HBED was decreased. The survival fraction was calculated and a significant decrease in survival of MiaPaCa-2 cells was obtained, with the strongest and statistically significant inhibition observed when the drugs were combined (Figure 5A, *right*). Figure 5B (*left* and *right*) shows that Mito-Met and HBED also significantly decreased pancreatic cancer cell colony formation, except that Mito-Met was effective at micromolar concentrations as compared with Met, present at millimolar levels. Mito-Met is nearly 1,000-fod more effective than Met.

### Effect of iron chelators and Met analogs on mitochondrial complex I activity, ROS formation

**Figure 6:**
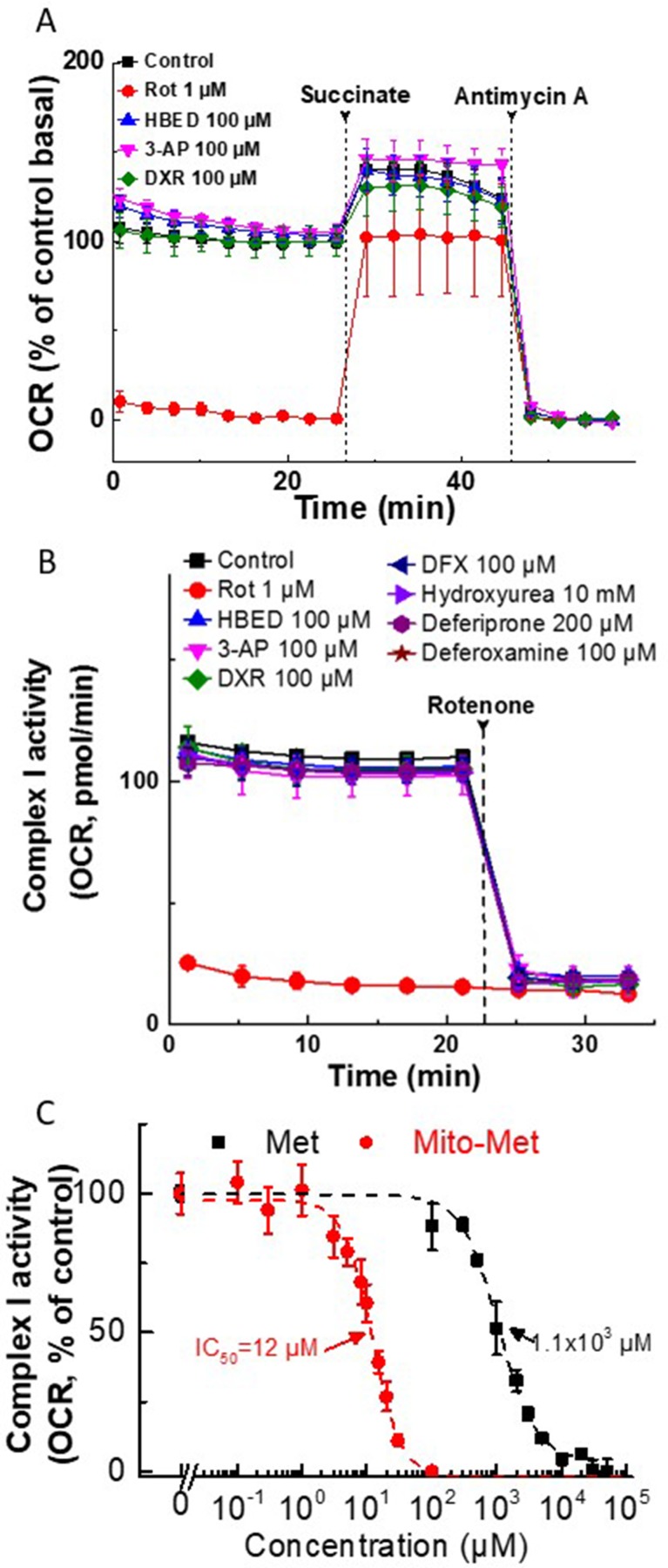
Effect of iron chelators and Met/Mito-Met on mitochondrial complex I activity in MiaPaCa-2 and MDA-MB- 231 cells. (**A**) Permeabilized MiaPaCa-2 cells were assayed in medium containing 10 mM pyruvate and 1.5 mM malate (substrates for complex I) in mannitol and sucrose (MAS) buffer. Either rotenone (Rot, complex I inhibitor) or iron chelators were added acutely and the OCR was assayed immediately. Both succinate (substrate for complex II, 10 mM) and antimycin A (complex III inhibitor, 20 μM) were injected, as indicated by the dashed lines. The mitochondrial complex I-dependent oxygen consumption was monitored as OCR trace shown before succinate injection. The three iron chelators tested showed no effects on complex I activity at up to 100 μM concentration. Rotenone was used as a positive control. (**B**) Permeabilized MDA-MB-231 cells were assayed in medium containing 10 mM pyruvate and 1.5 mM malate as panel A in MAS buffer. Either rotenone (Rot, complex I inhibitor) or iron chelators were added acutely, and OCR was assayed immediately. The complex I inhibitor Rot (1 μM) was injected where indicated. The mitochondrial complex I-dependent oxygen consumption was monitored as shown by the OCR trace. The iron chelators tested showed no effects on complex I activity at up to a 100 μM–10 mM concentration. (**C**) The effect of Met and Mito-Met on the activity of mitochondrial complex I in human breast cancer cells. MDA-MB-231 cells were pretreated with Met or Mito-Met for 24 h. The mitochondrial complex I OCRs are plotted against the concentration of Met or Mito-Met. Dashed lines represent the fitting curves used for determination of the IC_50_ values. OCR was measured as in (B).

Because iron chelators and Met analogs effectively induced synergistic inhibitory effects on cancer cell proliferation, we tested their effects under these conditions on mitochondrial function (Figure 6A, 6B). As reported previously [7], both Met (millimolar concentration) and Mito-Met (micromolar concentration) inhibited the mitochondrial complex I-mediated oxygen consumption rate (OCR) in MDA-MB-231 cells (Figure 6C). However, at 100 μM concentration, equal to or higher than the one that exacerbated the antiproliferative effects of Met and Mito-Met, the iron chelators, HBED, 3-AP, or DXR, did not inhibit mitochondrial complex I-dependent OCR in MiaPaCa-2 and MDA-MB-231 cells (Figure 6A, 6B). It was previously reported that, at much higher concentrations (1 mM), DFO inhibited mitochondrial respiration in neuroblastoma cells [19]. From these results, we conclude that iron chelators and Met/Mito-Met do not induce a synergistic inhibition of OCR in cancer cells.

**Figure 7:**
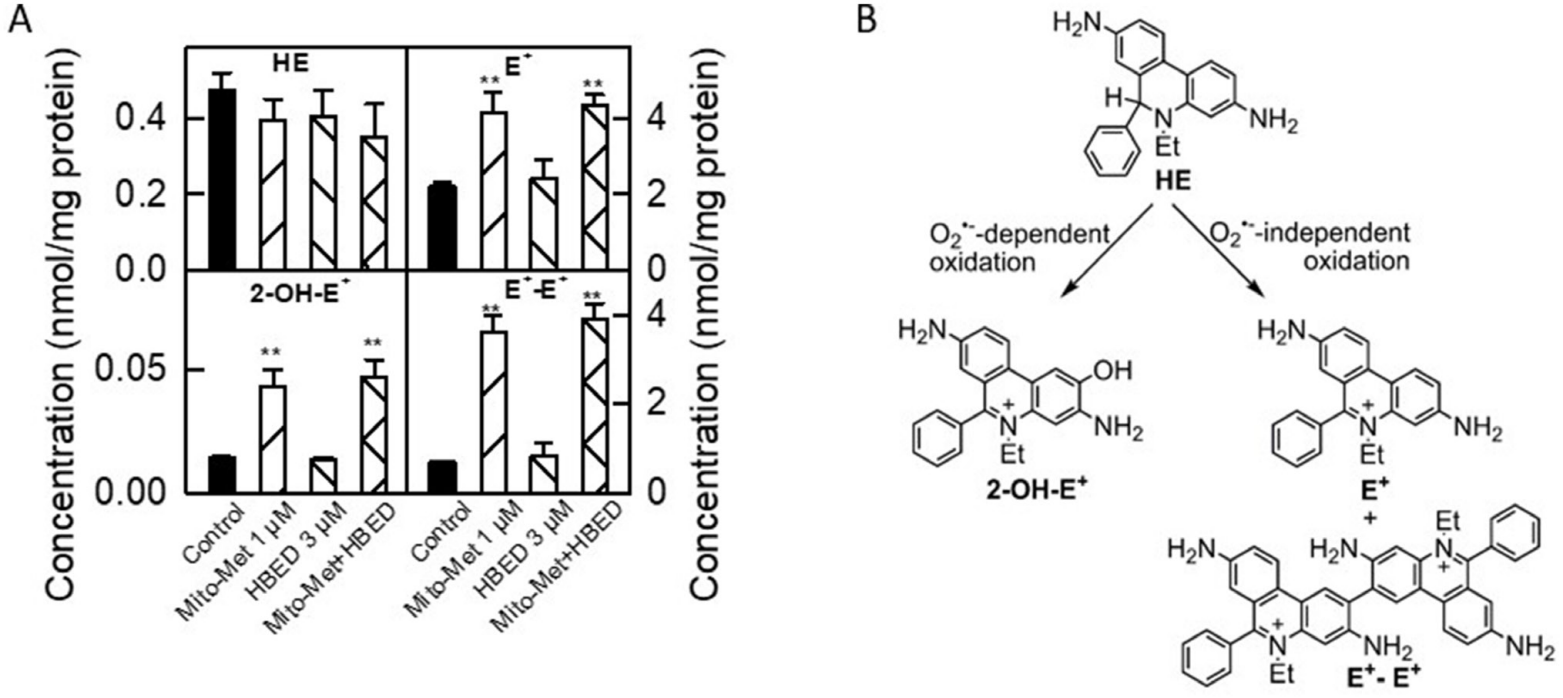
Characterization of intracellular oxidants induced by Mito-Met and HBED in MiaPaCa-2 cells. (**A**) MiaPaCa-2 cells were treated with HBED (3 μM) or Mito-Met (1 μM) alone or in combination for 24 h. Next, the cells were washed free of excess Mito-Met and HBED and treated with the ROS probe, HE (10 μM), for 60 min. Cells were then lysed and analyzed by HPLC. Bar graphs show the results of quantitative analysis of intracellular levels of HE probe and oxidation/hydroxylation products formed from HE. (**B**) A schematic representation of the superoxide-dependent and independent pathways of HE oxidation. 2-hydroxyethidium (2-OH-E^+^) is a superoxide-specific product, E^+^ is a nonspecific oxidation product, and E^+^-E^+^ is a product of one-electron oxidation of the probe.

The superoxide (O_2_^·–^)-specific probe, hydroethidine (HE), was used to detect Mito-Met-induced superoxide generated by inhibiting complex I in MiaPaCa-2 cells (Figure 7) [20]. In addition to O_2_^·–^, Mito-Met induced peroxidatic oxidants as determined by monitoring the increase in nonspecific oxidation product, ethidium cation (E^+^), and peroxidase-catalyzed formation of dimers (diethidium [E^+^-E^+^]) (Figure 7A, 7B). Inclusion of the iron chelator, HBED, did not affect Mito-Met-induced formation of these reactive oxygen species (ROS)-specific and nonspecific oxidation products of HE. These results rule out the possible role of ROS as a potential mechanism for the synergistic antiproliferative effect of iron chelators and Met analogs.

### Role of hypoxia on Met/Mito-Met- and iron-chelator-induced effects

**Figure 8:**
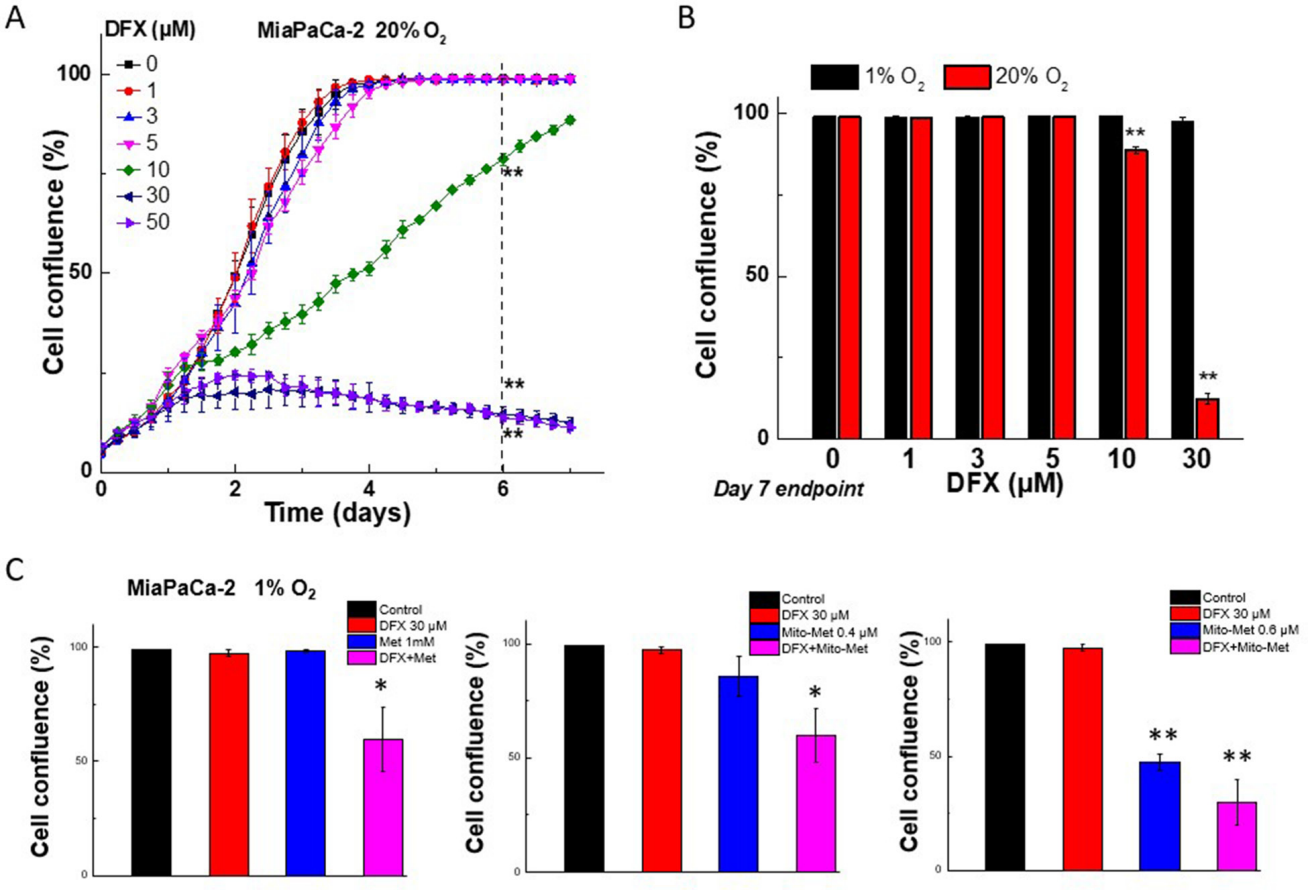
Effect of normoxia and hypoxia on the antiproliferative effects of DFX iron chelator, Met, and Mito-Met against MiaPaCa-2 cells. (**A**) MiaPaCa-2 cells cultured at 20% oxygen were treated with DFX and cell growth was monitored continuously up to 7 days. (**B**) The comparison of the effects of DFX on cell proliferation under hypoxia (1% O_2_) and normoxia (20% O_2_) at the same treatments time point. (**C**) Effect of Met analogs and iron chelator, DFX, on MiaPaCa-2 cell proliferation under hypoxia (1% O_2_) condition. Cells were treated with DFX and Met (C*, left*) or Mito-Met (C*, middle and right*) independently and together, as indicated, and cell growth was shown as cell confluence. Data shown are the mean ± SD (*n* = 4). (^*^P < 0.05, ^**^P < 0.05)

It was reported that the effect of iron chelator DFX observed at low oxygen levels is not the same as those obtained under high oxygen levels in human glioblastoma cells [21]. For example, the iron chelator DFX inhibited the proliferation of glioblastoma cells under normoxic conditions; however, this effect was reversed under low oxygen tension (3% O_2_) [21]. Thus, we investigated the effect of hypoxia (1% O_2_) on the antiproliferative potency of DFX in MiaPaCa-2 cells. As shown in Figure 8A, DFX (30 μM) significantly inhibited cell proliferation under normoxia (20% O_2_). However, under hypoxic conditions (1% O_2_), the inhibitory effect on cell proliferation was abolished (Figure 8B). This is consistent with the previous report using glioblastoma cells [21]. Conversely, when combined, both Met and DFX as well as Mito-Met and DFX, inhibited cell proliferation even under 1% O_2_ (Figure 8C). This is consistent with our previous study, which showed that hypoxic conditions did not reverse the inhibitory effect of Met or Mito-Met in pancreatic cancer cells [7].

## DISCUSSION

In this work, we discovered that several FDA-approved iron chelators can synergistically enhance the antiproliferative potency of relatively nontoxic drugs, Met and Mito-Met mitochondria-targeted analog, in pancreatic and triple-negative breast cancer cells. These findings could have significant clinical and translational impact on the application of Met and Mito-Met and related analogs in cancer chemotherapy and radiation therapy.

### Iron chelators and anticancer effects

The role of iron and ROS in cancer tumor proliferation and tumor growth and metastasis is paradoxical [22, 23]. Tumor cells require more iron than normal cells to sustain increased demands for energy, protein function, and DNA synthesis. For example, the activity of ribonucleotide reductase (RR), a key enzyme that catalyzes the conversion of ribonucleotides to deoxyribonucleotides, depends on the presence of iron in its M2 subunit [24, 25]. Since the RRM2 subunit in mammalian cells has a half-life of ~4 hours, a steady supply of iron is needed for RR activity. Limitation of iron availability to RRM2 inhibits RR activity and blocks DNA synthesis. Tumor cells exhibit increased expression and activity of RR as well as increased expression of the transferrin receptor at the cell surface, enabling enhanced cellular uptake of iron [26]. Iron chelators exert antiproliferative effects *in vitro* and *in vivo* on various cancers through inhibition of RR [27, 28]. Alternatively, inhibiting transferrin-receptor-dependent iron uptake also results in inhibition of RR and of tumor cell proliferation [29, 30]. We tested the effect of hydroxyurea (RR inhibitor) and Met analogs on pancreatic cancer cell proliferation (Supplementary Figure 4). Results indicate that there was no synergistic effect on cell proliferation, suggesting that RR inhibition is not responsible for the synergistic effect observed with iron chelator and Met analogs.

Enhanced intracellular iron can increase lipid peroxidation via the Fenton-type reaction and release of electrophilic metabolites that can stimulate apoptosis or ferroptosis of cancer cells [31]. Overexpression of glutathione-dependent peroxidase that supports the detoxification of lipid hydroperoxides inhibits ferroptosis-mediated cancer cell death [32]. Iron chelators could potentially thwart ferroptosis by inhibiting the Fenton-type reaction and exerting pro-tumorigenic effects [33]. Thus, the ultimate effect of iron chelators may depend on several factors, including the timing of treatment, activity of redox enzymes, and type of cancer cells.

Recently, it was reported that clinically relevant antioxidant drugs (vitamin E and N-acetyl cysteine) enhance metastasis of tumors by decreasing ROS [34]. Iron chelation did not significantly affect Mito-Met-induced ROS formation in tumor cells (Figure 7). Thus, the combination of Met/Mito-Met and iron chelators is unlikely to enhance tumor metastasis, although this must be proven in *in vivo* tumor xenografts.

### Metformin and analogs and antitumor effects

Metformin is weakly cationic and targets mitochondria, inhibiting complex I-dependent respiration in cancer cells [35]. However, a high concentration of Met is required to inhibit cancer cell respiration and proliferation [7]. The intracellular uptake of Met is enhanced in cancer cells exhibiting increased expression of the organic cation transporter [36]. Ovarian cancer cells with a downregulated organic cation transporter are resistant to Met toxicity [37]. In contrast to Met, Mito-Met is nearly 1,000-times more effective in inhibiting pancreatic cancer cell proliferation [7]. Mito-Met is effective at low micromolar levels [7]. The cell permeability of Mito-Met is increased because of the presence of a long alkyl chain and the delocalized lipophilic cationic moiety, TPP^+^ [7]. The mitochondrial membrane potential in cancer cells is more negative as compared with normal cell mitochondria, and the presence of permanent positive charge is a major driving force for mitochondrial accumulation [38]. Mito-Met inhibits complex I, inducing ROS and AMP-activated protein kinase (AMPK) activation and resulting in enhanced signaling and activity of antiproliferative processes. At the concentrations used in this study, Mito-Met had no effect on the growth of nontumorigenic cells [7].

### Synergistic effect of iron chelators on antitumor drugs

Synergism between the iron chelator, DFX, and conventional cytotoxic chemotherapeutics (*e.g.*, doxorubicin, cyclophosphamide, and cisplatin or carboplatin) in the treatment of triple-negative breast cancers has previously been reported [39]. Therapeutic targeting of iron metabolism for breast cancer treatment was reported many years ago [40, 41]. In this work, we demonstrate the ability of iron chelators to synergize with relatively nontoxic and tumor-cell-specific antiproliferative agents (Met and Mito-Met). Met potentiated the antitumor effects of doxorubicin in pancreatic and breast cancer cells [42]. Thus, the combined use of conventional chemotherapeutics, iron chelators, and Met or Mito-Met is likely to be a potent antitumor cocktail. Radiation treatment exacerbated the antiproliferative effects of Met and Mito-Met in pancreatic cancer cells [7]. It would be of interest to investigate the effect of radiation on the combined effects of Met analogs and iron chelators. This proposal should, however, be tested both *in vitro* and *in vivo* prior to being translated to the clinic.

### Stimulation of autophagy by iron chelators and Mito-Met in cancer cells

Iron chelators and mitochondria-targeted agents have been reported to induce autophagy in several cancer cells [39, 43–46]. The iron chelator DFO was shown to inhibit TRAIL-mediated apoptosis *via* induction of autophagy flux, and chloroquine, an inhibitor of autophagy, blocks DFO-mediated tumor cell death *via* the autophagy pathway [44]. The anticancer agent with an iron chelating ability (di-2-pyridylketone-4,4-dimethyl-3-thiosemicarbazone [Dp44mT]) activated autophagy kinase, Unk-51-like kinase, in tumor cells [45]. Recently, Mito-Met and another mitochondria-targeted agent (Mito-CP) were shown to stimulate mitophagy (mitochondrial autophagy) in colon cancer cells [46]. It is conceivable that iron chelators and Met analogs synergistically inhibit cell proliferation *via* enhanced stimulation of mitophagy in cancer cells.

### Potential cardioprotective effect by Met analogs and iron chelators

Another aspect of Met analogs and iron chelators therapies of cancers is that both Met and DXR have exhibited cardioprotective effects in human and animal studies [47–49]. Thus, it is plausible that the combined use of Met analogs and iron chelators (*e.g.*, DFX) will not only decrease the tumor proliferation (as discussed in this work) but could potentially protect cardiomyocytes against oxidative damage. Heart problems in doxorubicin-treated cancer patients do not manifest until many years post chemotherapy [50–52]. Children with leukemia that are treated with doxorubicin develop cardiotoxicity after they reach adulthood, and the onset and severity of the toxicity were decreased when treated with the iron chelator, DXR. In other studies, treatment with Met was shown to afford cardioprotection [53].

### Could copper chelators also induce a synergistic antiproliferative effect with Met analogs in cancer cells?

Both iron and copper are important for cancer cell proliferation [54]. Whereas iron chelators (*e.g.*, Triapine) show promise in cancer therapy, copper chelators such as penicillamine revealed an antitumor effect [54]. It has been reported that genetic perturbation of copper or decreasing copper levels with specific copper chelators inhibits growth of drug-resistant melanoma cells [55, 56]. In this regard, it is of interest to note that metformin itself is a moderate to strong copper chelator [57], and this effect was reported to play a role in cancer cell killing [58]. Based on these results, it seems plausible that copper chelators combined with mitochondrial antitumor drugs may induce a synergistic antiproliferative effect in cancer cells. Clearly, detailed studies with copper chelators and metformin analyses are warranted.

## MATERIALS AND METHODS

### Chemicals

Mito-Met was synthesized and purified as reported previously [7]. Iron chelators were purchased from Sigma-Aldrich (St. Louis, MO, USA).

### Cell lines

Human MiaPaCa-2 pancreatic cancer and wild-type and brain homing MDA-MB-231 breast cancer cells were purchased from ATCC (Manassas, VA, USA). All cells were kept frozen in liquid nitrogen and were used within 20 passages after thawing. The cells were cultured under standard conditions (37°C and 5% CO_2_) in Dulbecco’s Modified Eagle Medium (Thermo Fisher Scientific, Waltham, MA, USA, Catalog No. 11965) supplemented with 10% fetal bovine serum, 100 units/ml penicillin, and 100 μg/ml streptomycin (Thermo Fisher Scientific, Waltham, MA, USA).

### Cell proliferation and clonogenic assays

Cell proliferation was measured using a probe-free, noninvasive cellular confluence assay by the IncuCyte Live Cell System (IncuCyte FLR, Essen BioScience, Ann Arbor, MI, USA), as described previously [7]. Changes in cell confluence were used as the surrogate marker of cell proliferation.

For clonogenic assay, cells were seeded as indicated in six-well plates and treated with Mito-Met or Met for 24 h. The plates were placed within the incubator and the cell culture media changed every 3–4 days until the control (untreated) cells formed sufficiently large clones. The cell survival fractions were calculated as previously reported [7].

### Mitochondrial function measurements

The mitochondrial function was measured in real time using a Seahorse XF96 Extracellular Flux Analyzer (Agilent, North Billerica, MA, USA). Assays in intact cells were performed as reported previously [7]. The OCR derived from mitochondrial complex I and complex II activities was measured in the presence of different mitochondrial substrates, *e.g.*, pyruvate/malate for complex I and succinate for complex II. Rotenone, malonate, and antimycin A were used as specific inhibitors of mitochondrial oxygen consumption at complexes I, II, and III, respectively.

### ROS and oxidation products

ROS were measured using the probe, HE. A stock solution of HE (20 μM) was prepared in deoxygenated dimethyl sulfoxide and stored in the dark at −80°C until use. Previously, we showed that a global analysis of oxidation products derived from HE provides a better picture of oxidants generated intracellularly. To this end, we characterized the various oxidation products from HE using the appropriate standards [65, 66]. The hydroxylated oxidation product, 2-OH-E^+^, formed from HE reaction with O_2_^·–^ was prepared by reacting HE with Fremy’s salt [65]. The nonspecific, two-electron oxidation product of HE, E^+^ (bromide salt), was purchased from Sigma-Aldrich (St. Louis, MO, USA). The dimeric product (E^+^-E^+^) was prepared by oxidizing HE with excess ferricyanide [65]. All standards were purified by high-performance liquid chromatography (HPLC).

### HPLC and LC-MS/MS

HPLC and liquid chromatography–mass spectrometry (LC–MS/MS) analyses were performed as previously described [7]. Briefly, cells were grown in 10 cm dishes and incubated with Mito-Met, Met, or iron chelator HBED for 24 h in full media. HPLC analysis was performed using Kinetex C_18_ column.

## CONCLUSIONS

In this study, we report that the combined use of iron chelators, some of which are FDA-approved drugs, and Met analogs synergistically inhibits proliferation of pancreatic and breast cancer cells. Iron chelators previously have been shown to inhibit cancer cell proliferation when combined with conventional cytotoxic chemotherapeutics [7, 39, 59]. OXPHOS is emerging as an important target in cancer therapy [60]. The recent data suggest that iron chelators could exacerbate the antiproliferative effect of OXPHOS inhibitors in cancer cells. Here, we show that iron chelators potently sensitize and exacerbate the antiproliferative effects of relatively nontoxic mitochondria-targeted drugs in cancer cells. Although the exact molecular mechanism of action has not been determined, it is conceivable that multiple cellular targets and several mechanisms (*e.g.*, cell cycle arrest and cell cycle dysregulation, disruption of mitochondrial redox signaling, topoisomerase inhibition and DNA damage, activation of tumor suppressor genes, endoplasmic reticulum stress, and autophagic pathways) are involved [61–64].

## SUPPLEMENTARY MATERIALS



## Abbreviations

2-OH-E^+^: 2-hydroxyethidium
AMPK: AMP-activated protein kinase
CI-Fa: Combination index-fraction affected
DFO: Deferoxamine
DFX: Deferasirox
Dp44mT: di-2-pyridylketone-4,4-dimethyl-3-thiosemicarbazone
DXR: Dexrazoxane
E^+^: Ethidium
E^+^-E^+^: Diethidium
HBED: N,N’-bis(2-hydroxyphenyl)ethylendiamine-N,N’-diacetic acid
HE: Hydroethidine
HPLC: High-performance liquid chromatography
LC–MS/MS: Liquid chromatography–mass spectrometry
MAS: Mannitol and sucrose
Met: Metformin
Mito-Met: Mito-metformin
O_2_^·–^: Superoxide
OXPHOS: Oxidative phosphorylation
ROS: Reactive oxygen species
RR: Ribonucleotide reductase
TPP^+^: Triphenylphosphonium cation
