# Supplementary material for: Synergistic inhibition of tumor cell proliferation by metformin and mito-metformin in the presence of iron chelators
Source: Oncotarget. 2019 May 28;10(37):3518–32. doi: 10.18632/oncotarget.26943 (PMC6544408; doi:10.18632/oncotarget.26943)
Supplement: Supplementary file 1 [file oncotarget-10-3518-s001.pdf]

Synergistic inhibition of tumor cell proliferation by metformin and mito-metformin in the presence of iron chelators

SUPPLEMENTARY MATERIALS

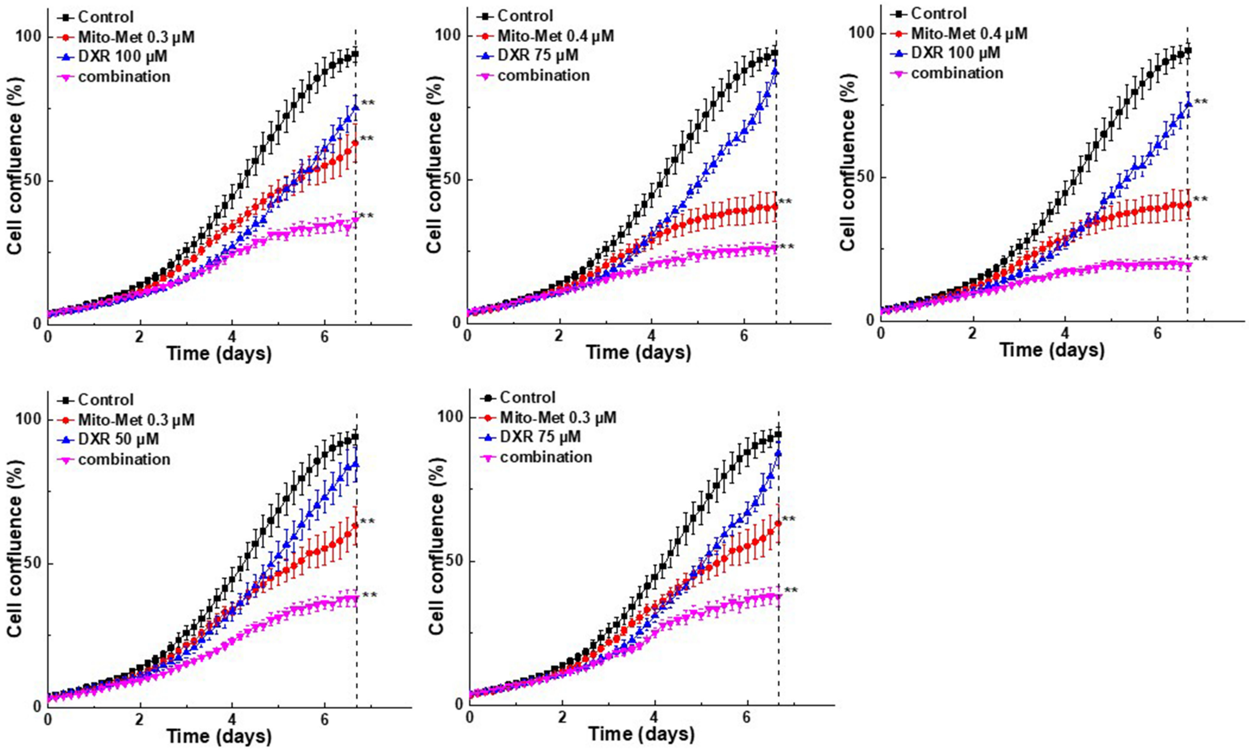

Supplementary Figure 1: Inhibition of cell proliferation by DXR and Mito-Met in pancreatic cancer cells, PANC-1. PANC-1 human pancreatic cancer cells were treated with DXR or Mito-Met independently and together, at different concentrations as indicated, and cell growth was monitored continuously. Data shown are the mean ± SD ($n = 4$). The dotted vertical lines indicate the time points at which the levels of significance were calculated ($^{**}P < 0.01$).

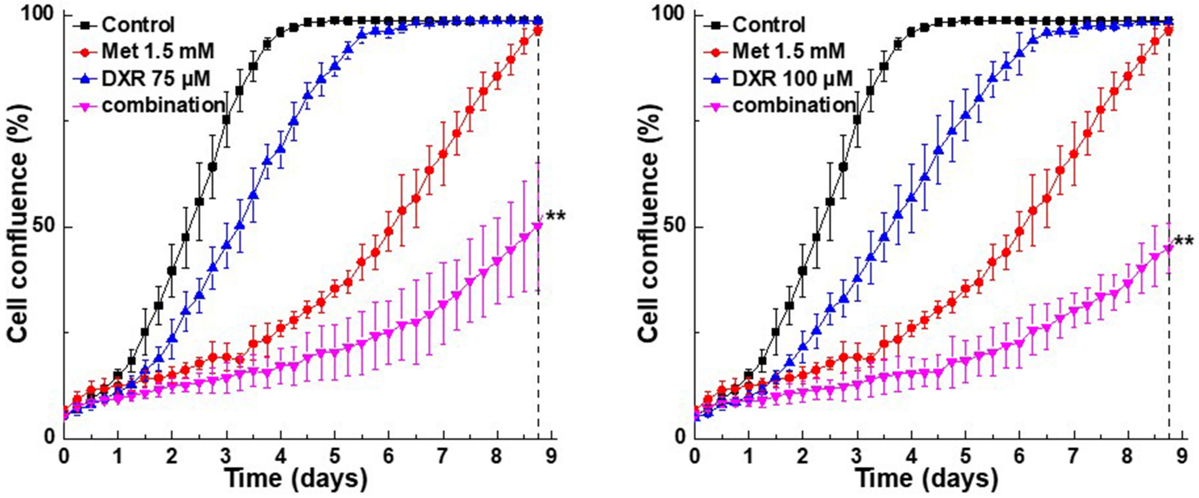

Supplementary Figure 2: Inhibition of cell proliferation by DXR and Mito-Met in pancreatic cancer cells, AsPC-1. AsPC-1 human pancreatic cancer cells were treated with DXR or Mito-Met independently and together, as indicated, and cell growth was monitored continuously. Data shown are the mean ± SD ($n = 4$). The dotted vertical lines indicate the time points at which the levels of significance were calculated ($^{**}P < 0.01$).

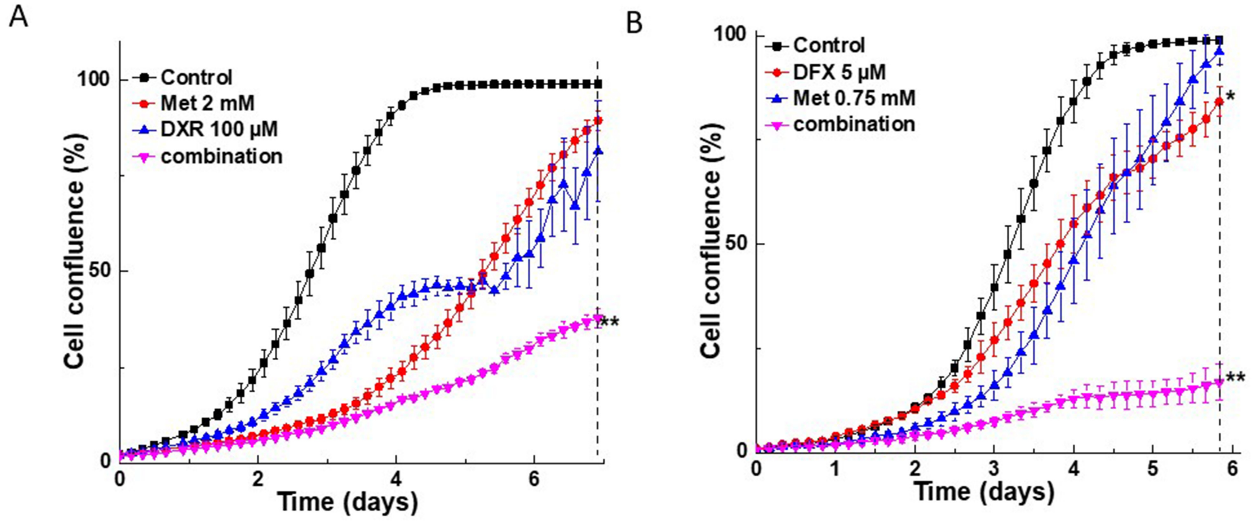

Supplementary Figure 3: Inhibition of cell proliferation by DXR or DFX in combination with Met in mouse pancreatic cancer cells, FC1242. Mouse pancreatic cancer cells were treated with DXR (**A**) or DFX (**B**) and Met independently and together, as indicated, and cell growth was monitored continuously. Data shown are the mean ± SD ($n = 4$). The dotted vertical lines indicate the time points at which the levels of significance were calculated ($^{**}P < 0.01$).

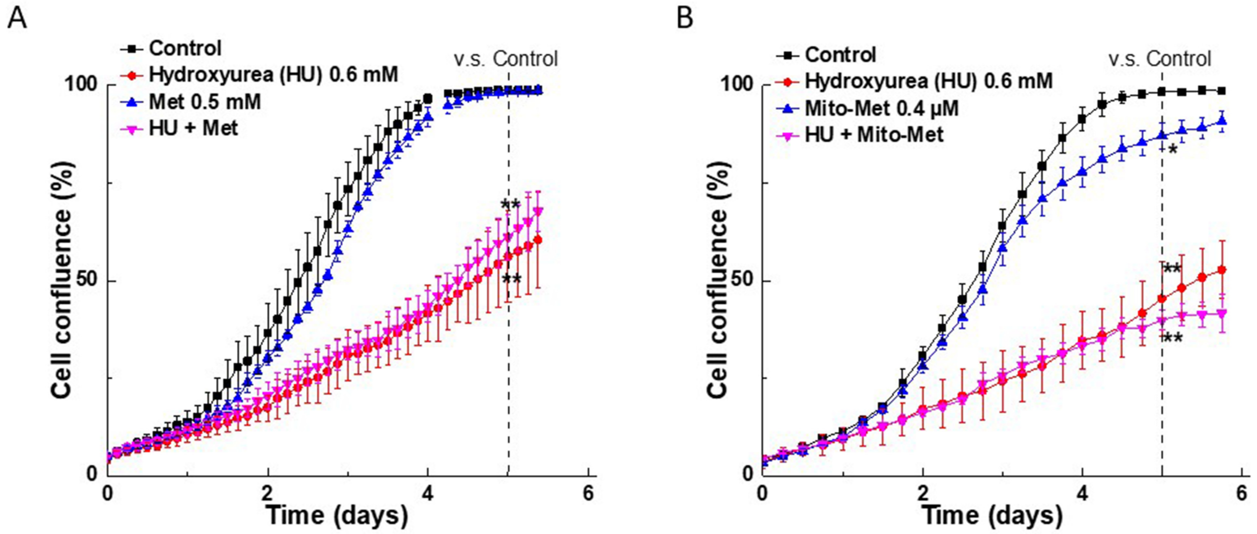

Supplementary Figure 4: Inhibition of cell proliferation by hydroxyurea and Met analogs in pancreatic cancer cells, MiaPaCa-2. Cells were treated with hydroxyurea and Met (**A**) or hydroxyurea and Mito-Met (**B**) independently and together and cell growth monitored continuously. Data shown are the mean ± SD ($n = 4$). The dotted vertical lines indicate the time points at which the levels of significance were calculated ($^{**}P < 0.01$).
